# Retinal vascular occlusions during COVID-19: an epidemiological survey

**DOI:** 10.3389/fopht.2026.1804194

**Published:** 2026-04-27

**Authors:** Andrea Montesel, Yan Guex-Crosier, Chiara M. Eandi

**Affiliations:** 1 Department of Ophthalmology, Fondation Asile des Aveugles, Jules-Gonin Eye Hospital, University of Lausanne, Lausanne, Switzerland; 2 Department of Surgical Sciences, University of Torino, Torino, Italy

**Keywords:** COVID-19, pandemic, retinal vascular occlusion, RVO, SARS-CoV-2, thrombosis

## Abstract

**Purpose:**

To evaluate whether the COVID-19 pandemic was associated with changes in the incidence of retinal vascular occlusions.

**Methods:**

We performed a retrospective cohort study at the Jules-Gonin Eye Hospital (Lausanne). All new diagnoses of central retinal vein occlusion (CRVO), branch retinal vein occlusion (BRVO), central retinal artery occlusion (CRAO), and branch retinal artery occlusion (BRAO) between January 1, 2019 and December 31, 2020 were included. Regional COVID-19 case counts were obtained from public health records. Incidence rate ratios (IRR) comparing 2019 cases versus 2020 were calculated using official population data. Pearson correlation was used to explore temporal associations between monthly COVID-19 cases and retinal vascular events. The study period largely reflects natural infection exposure, as COVID-19 vaccination in Switzerland began on December 23, 2020.

**Results:**

In 2019, 66 retinal vein occlusions (38 CRVO, 28 BRVO) and 20 arterial occlusions (9 CRAO, 11 BRAO) were recorded. In 2020, 49 retinal vein occlusions (34 CRVO, 15 BRVO) and 21 arterial occlusions (14 CRAO, 7 BRAO) were recorded. The IRR for 2020 versus 2019 was 0.73 for vein occlusions (95% CI 0.51–1.05; *p*=.093) and 1.04 for arterial occlusions (95% CI 0.57–1.90; *p*=.896). Monthly analysis showed no significant temporal association between COVID-19 incidence and retinal vascular events (*p*=.08).

**Conclusions:**

In this regional study, we did not observe an increase in retinal vascular occlusive events during the early phase of the COVID-19 pandemic. While biologically plausible mechanisms have been proposed, these population-level trends do not support a strong association.

## Highlights

What is known

- COVID-19 has been associated with vascular and thromboembolic complications, and retinal vascular occlusions have been described in COVID-19 patients.
- There is limited population-level data evaluating whether COVID-19 has modified the incidence of retinal vascular events.

What is new

- A retrospective cohort study was conducted in the Canton of Vaud (Switzerland), comparing the hospital-based incidence of retinal vascular occlusions before and during the pandemic.
- Our findings suggest that the pandemic did not lead to an increased burden of retinal vascular events, challenging concerns based on biological plausibility and previous reports.

## Introduction

The COVID-19 pandemic, caused by the SARS-CoV-2 virus, has raised significant concern regarding its systemic vascular and thromboembolic complications. In particular, the prothrombotic state and endothelial dysfunction associated with SARS-CoV-2 infection have prompted investigation into potential retinal vascular events (RVE, including retinal vein occlusions, RVO, and retinal artery occlusions RAO). Although numerous case reports have documented both retinal vein and artery occlusions in patients with COVID-19 (1), robust population-based data assessing the broader impact of the pandemic on the incidence of RVE remain scarce.

This study aimed to examine trends in the hospital-based incidence of RVE in the Canton of Vaud, Switzerland - a region significantly impacted by the pandemic. We conducted a retrospective cohort study at the Jules-Gonin Eye Hospital (Lausanne, Switzerland), the main regional tertiary referral eye centre, which provides the primary ophthalmic emergency service for acute retinal vascular occlusions in the region.

The study period covered January 1, 2019 to December 31, 2020. Public health records provided monthly COVID-19 cases in the Canton of Vaud, with the first confirmed case reported on February 27, 2020 (2). Importantly, the first COVID-19 vaccine dose in Switzerland was administered on December 23, 2020, allowing us to focus on a population exposed only to natural infection during the study period, without the potential confounding effects of vaccination.

Cases were identified through systematic searches of the institutional electronic medical records using diagnostic keywords corresponding to central retinal vein occlusion (CRVO), branch retinal vein occlusion (BRVO), central retinal artery occlusion (CRAO), and branch retinal artery occlusion (BRAO). Only incident (new) diagnoses made between January 1, 2019 and December 31, 2020 were included. Follow-up visits, recurrent events in previously diagnosed eyes, and miscoded entries were excluded after manual chart review. All included cases were reviewed and confirmed by a senior retinal specialist (AM) based on clinical examination and multimodal imaging (fundus photography, OCT, and when available fluorescein angiography), ensuring diagnostic accuracy and correct subtype classification. Cases were then stratified by month and aggregated; formal approval was obtained from the local ethics committee (CER-VD 01612). Pearson’s correlation coefficient was used to assess the relationship between monthly RVE and COVID-19 case-counts. Incidence Rate Ratios (IRR) for retinal vascular occlusions were calculated comparing 2020 to 2019, using the corresponding population data from official public health records, with 2019 serving as the reference year (3).

## Results

**Figure 1:**
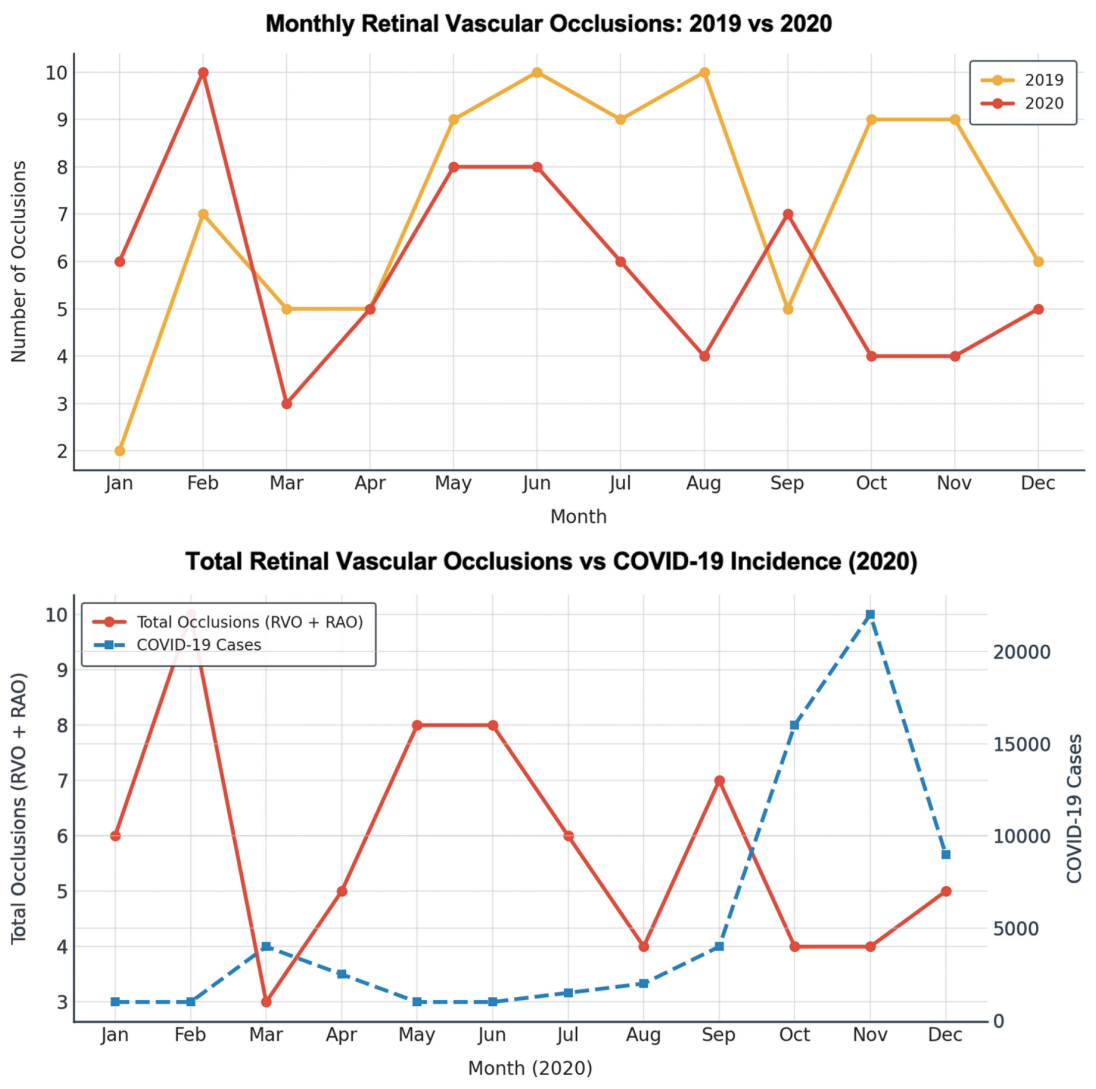
Monthly retinal vascular occlusions in 2019 and 2020, and association with COVID-19 incidence in 2020. Upper panel: monthly distribution of total retinal vascular occlusions (RVO + RAO) in 2019 (orange) and 2020 (red). Lower panel: monthly retinal vascular occlusions (red, left y-axis) compared with reported COVID-19 cases (blue, right y-axis) during 2020.

In 2019, 66 cases of vein occlusions (38 CRVO and 28 BRVO) were recorded, and 20 cases of arterial occlusions (9 CRAO, 11 BRAO). In 2020, 49 cases of vein occlusions (34 CRVO and 15 BRVO) were recorded, and 21 cases of arterial occlusions (14 CRAO, 7 BRAO) (**Figure 1**).

The IRR for 2020 compared to 2019 was 0.73 for retinal vein occlusions (95% CI 0.51 - 1.05, *p = .*093) and 1.04 (95% CI 0.57 - 1.90, *p* = .896) for the arterial ones (**Table 1**). Monthly analysis showed no clear temporal association between COVID-19 and retinal vascular events. The Pearson correlation coefficient between RVE and COVID-19 cases was *r* = −0.52 (*p* = .08), not reaching statistical significance.

**Table 1:** Retinal vascular occlusions (2019 vs 2020).

Type	2019 cases	2020 cases	IRR (2020 vs 2019)	95% CI & *p*-value
**Total vein occlusions**	**66**	**49**	**0.73**	**95% CI 0.51 - 1.05, *p* = .093**
CRVO	38	34		
BRVO	28	15		
**Total arterial occlusions**	**20**	**21**	**1.04**	**95% CI 0.57 - 1.90, *p* = .896**
CRAO	9	14		
BRAO	11	7		
**Total retinal vascular occlusions**	**86**	**70**	**0.81**	**95% CI 0.59 - 1.12, *p* = .201**

Distribution of retinal vascular occlusions in 2019 and 2020, stratified by type (central and branch vein occlusion; central and branch arterial occlusion). The table reports absolute case numbers for each year together with hospital-based incidence rate ratios (IRR) comparing 2020 vs 2019, with corresponding 95% confidence intervals and *p* values.

## Discussion

Our study found a modest decrease in retinal vascular events in 2020 compared to 2019, despite the onset of the COVID-19 pandemic. This contrasts with previous reports suggesting a possible increase in thrombotic events during SARS-CoV-2 infection (1, 4).

Several factors could explain the observed decline. Changes in healthcare-seeking behaviour, delayed consultations, or underdiagnosis during lockdowns may have reduced the detection of RVE, particularly for mild or asymptomatic occlusions. Alternatively, the pandemic may not have had a significant direct impact on retinal vascular events incidence in the general population. While systemic inflammation and endothelial injury are known effects of COVID-19, our data do not indicate an association between infection rates and new RVE.

Our findings are consistent with a prior study from South Korea - the only nationwide, population-based retrospective cohort study reported in the literature - which found no increase in the incidence of arterial occlusions during the COVID-19 pandemic (5). In contrast, the authors reported an increase in the incidence of venous occlusions during the same period, a trend attributed to a rise in metabolic diseases rather than to COVID-19 infection or vaccination. The main limitation of this study is that our analysis is based on cases captured at the region’s main tertiary referral and emergency ophthalmology centre; therefore, the results should be interpreted as hospital-based event rates rather than strict population-based incidence. While some milder cases may have been managed in private practice, referral pathways were unlikely to shift case capture towards the private sector during the pandemic; if anything, reduced outpatient activity may have resulted in fewer cases being managed outside hospital-based services. Moreover, given the rarity of retinal vascular occlusive disease and the inherently limited case numbers at a single centre, this study should be considered exploratory and hypothesis-generating; no formal power analysis was performed. Other limitations include the retrospective design and the inability to adjust for individual comorbidities.

In conclusion, this regional study did not observe an increase in retinal vascular events during the first year of the COVID-19 pandemic. While biologically plausible mechanisms exist, population-level trends do not support a strong association. Future studies should explore individual-level risk factors and multi-year trends to clarify potential links between COVID-19 and retinal vascular disease.

## Data Availability

The raw data supporting the conclusions of this article will be made available by the authors, without undue reservation.
